# Public Health Approach in the Elimination and Control of Cervical Cancer: A Review

**DOI:** 10.7759/cureus.44543

**Published:** 2023-09-01

**Authors:** John Kessellie Jallah, Ashish Anjankar, Francis A Nankong

**Affiliations:** 1 Department of Biochemistry, Jawaharlal Nehru Medical College, Datta Meghe Institute of Higher Education and Research, Wardha, IND; 2 Department of Science and Technology, Jawaharlal Nehru Medical College, Datta Meghe Institute of Higher Education and Research, Wardha, IND

**Keywords:** cervical carcinoma, human papillomavirus-immunization, health disparities, human immunodeficiency virus (h.i.v.), cervical cancer screening and therapy

## Abstract

Public health experts worldwide have emphasized cervical cancer since it is a substantial global health burden primarily affecting women. This article thoroughly reviews the public health approach to eradicating and managing cervical cancer. The public health community seeks to lower the prevalence, morbidity, and mortality linked to this preventable disease by integrating primary prevention by means of vaccination against the human papillomavirus (HPV), secondary prevention using screening and early identification, and tertiary prevention through improved therapy and supportive care. In order to accomplish broad vaccination coverage and ultimately effectively prevent cervical cancer, it remains crucial to address obstacles to vaccine accessibility, reluctance, and fair distribution. Early identification and subsequent treatments depend greatly on cervical cancer screening programs. This study explores several screening methods, such as Papanicolaou (Pap) tests based on cytology and cutting-edge technologies like molecular assays and HPV detection. The detection of precancerous lesions and early-stage malignancies, permitting prompt treatment, has shown significant promise when integrating these technologies into coordinated population-based screening programs. The study also underlines the significance of addressing cervical cancer burden inequities, particularly in resource-constrained areas where access to preventative and curative care is constrained. Innovative and affordable methods for addressing marginalized groups are studied, including community-based outreach programs, mobile health technology, and local healthcare practitioners and community leaders in awareness campaigns. The research also examines improvements in cervical cancer treatment procedures, such as surgery, radiation, chemotherapy, and immunotherapy. It improves therapeutic efficacy and patient survival rates by incorporating various modalities into a multidisciplinary strategy. Highlighted palliative care and psychological support are crucial for patients who have advanced cervical carcinoma.

## Introduction and background

Considering that cervical carcinoma is a severe public health concern that has to be tackled, it is exceptionally accurate for small and medium-earning countries, where it is the fourth most common cancer affecting women. Cancer of the cervical cavity is more prevalent in women with HIV and has a high case-fatality rate. The World Health Organization (WHO) advocates for a threefold intervention strategy that includes immunization of a minimum of 90% of 15-year-old girls against human papillomaviruses (HPV), screening 70% of 35- and 45-year-old women utilizing a highly efficient test, and treating a minimum of 90% of pre-cancerous and aggressive tumour cancers that are discovered. This approach aims to eradicate four cases per 100,000 women worldwide [1-3]. Cervix cancer is the highest causative agent of death-related cancer in women globally, regardless of the advancement in diagnostic and medical procedures [4,5]. One of the key global factors influencing cervical carcinoma is HPV, the primary human source of risk for cervix cancer disease, which causes an infection spread through sexual contact. That is relatively common among people who participate in sexual activity and have multiple sex partners. Although multiple HPV strains exist, not all cause cervix cancer; the primary causes of cervical carcinoma are human papillomavirus types 16 and 18.

Globally, 604,127 new instances of cervix cancer were identified in 2020, with an estimated 341,831 women dying due to the disease. They occur in around 90% of cases in underprivileged and middle-income nations [6]. Cervical carcinoma is six times more likely to occur in women with HIV than women without HIV; an estimated 5% of cervical carcinoma occurrences are related to HIV. As a result, cervical neoplasm remains a worldwide health problem, especially in underdeveloped nations where Papanicolaou (Pap) test screening programs and vaccine campaigns are not generally available [7]. Incorporating the viral genome into a host chromosome is the primary cause of HPV-induced carcinogenesis. From this point forward, younger women in global regions are disproportionately affected by the contribution of HIV to cervix cancer. Appropriate medicines at different stages of life could contribute to lowering the alarming worldwide mortality rate from cancer of the cervix (age-standardized incidence in women: 13.3/100,000 in 2020) [1,8]. Recently, the significance of a global health strategy for cervical carcinoma control, eradication, and prevention has become widely recognized [9]. This strategy focuses on the requirement for coordinated, all-encompassing, and evidence-based solutions that address the various elements that affect the burden of the illness, such as social, economic, and environmental determinants of health.

## Review

Search methodology

The researchers performed a systematic study and comparative analysis of several papers about cervical cancer for this review article. The researchers also explore the recent state of preventing and managing cervical cancer and the newest diagnostic procedures and technological advancements in treating cervical cancer. Furthermore, the researchers initiated a comprehensive literature search in February 2023 using keywords such as "Cervical carcinoma", "Human papillomavirus-immunization", "Health disparities", "Human immunodeficiency virus", and "Cervical cancer screening and therapy", through various academic journals and search engines like Google Scholar, PubMed, and the International Journal of Gynecological Cancer.

**Figure 1:**
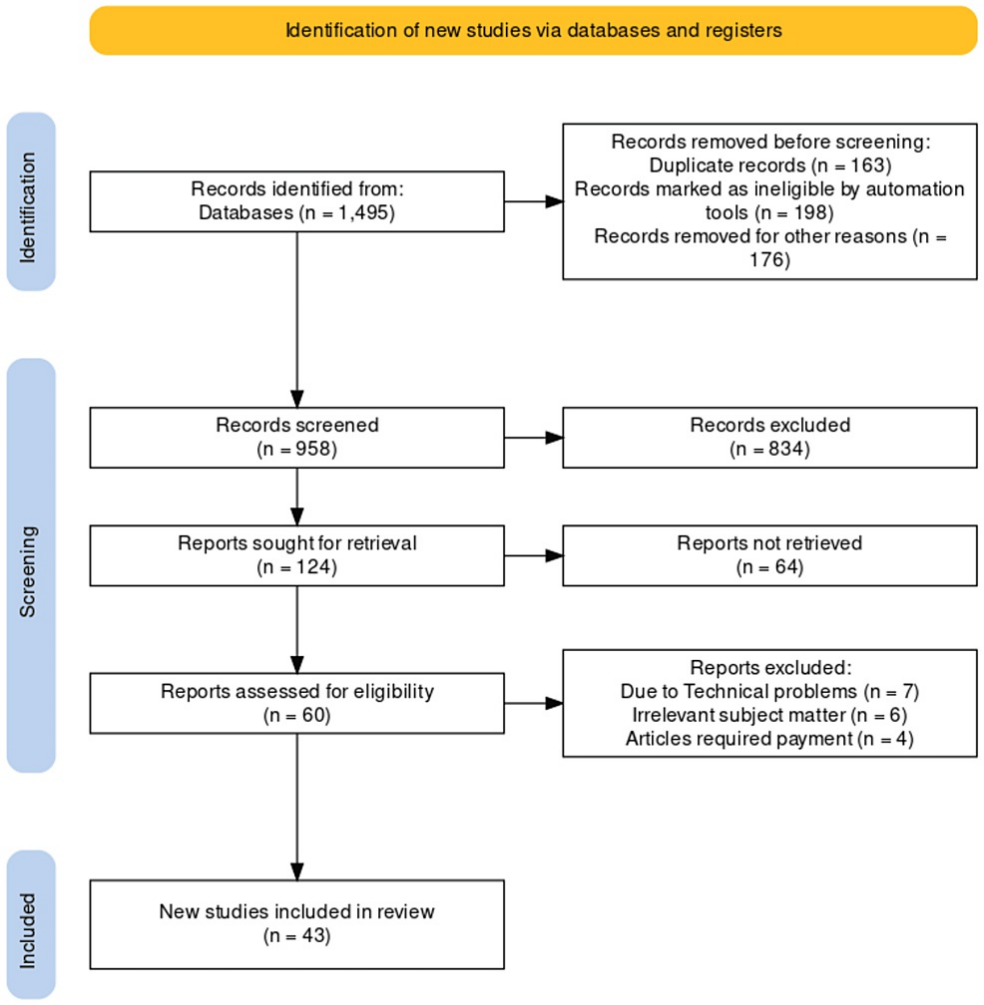
PRISMA flow diagram for search strategy and selection criteria Preferred Reporting Items for Systematic Reviews and Meta-Analyses

The selection criteria included in this study (Figure 1) are as follows: (1) cervical carcinoma; (2) human papillomavirus-immunization; (3) health disparities; (4) human immunodeficiency virus; (5) cervical cancer screening and therapy; (6) English language. The following were the exclusion criteria: (1) irrelevant subject matter; (2) technical issue; (3) article required payment; (4) non-English language. This review also explored advances in cervical cancer treatment procedures, such as surgery, radiation, chemotherapy, and immunotherapy.

Discussion

Cervical Cancer as a Public Health Concern

Regarding public health, cervical cancer is a significant concern, particularly for women with human immunodeficiency virus (HIV) as it weakens immunity, increasing the chance of infection with high-risk HPV, the primary factor causing cervix cancer [10,11]. However, it is crucial to understand that, despite having a somewhat decreased risk, women without HIV remain at risk of acquiring cervical cancer [12]. Implementing a public health strategy to prevent cervical cancer has received more attention recently. However, there are still many challenges, especially for low-income countries. This all-encompassing approach emphasizes early detection, prevention, and treatment, specifically focusing on vulnerable populations such as women with HIV [13].

Screening Women Without HIV

Cervical cancer prevalence and fatality rates in women without HIV have significantly decreased due to cervical cancer screening. A standard screening method is the Pap smear test, which includes looking for abnormalities in cervical cells [14]. It detects early cervical cancer and precancerous tumours, allowing prompt intervention and treatment. High-risk strains of human papillomavirus infection primarily contribute to chronic cervical cancer, a severe worldwide health problem. Globally, 604,127 new instances of cervix cancer were diagnosed in 2020, while the number of women who die from the disease is anticipated to reach 341,831. Around 90% of the time, they occur in underprivileged and middle-income nations [6].

On the other hand, effective screening programs may drastically reduce the risk of cervical carcinoma [15]. Dr Georgios Papanikolaou invented the Papanicolaou smear test, often called the Pap test, in 1940. By offering a quick and affordable way to find anomalies in cervical cells, it changed the screening process for cervical cancer. A healthcare professional obtains a sample of cervix cells for the Pap smear, which are inspected under a microscope for any cellular alterations or anomalies. Numerous studies have shown how well the Pap smear reduces the prevalence of mortality from cervical carcinoma. The United States Preventive Services Task Force (USPSTF) recommends Pap tests for cervical cancer detection every three years for women aged 21 to 65 years [10]. According to research, countries with solid screening programs have seen a considerable decrease in cervical cancer rates due to routine Pap smear examinations [16]. One of its key benefits is the Pap smear's ability to identify precancerous lesions like cervical intraepithelial neoplasia (CIN) before they develop into an invasive malignancy. Early identification makes it possible to carry out early therapies, including excision surgeries or ablation, which can stop the growth of cervix cancer. According to a thorough investigation and meta-analysis, depending on the screening interval and healthcare situation, the sensitivity of the Pap smear for identifying CIN2+ (moderate to severe cervical lesions) can range from 50% to 98% [17]. While significantly lowering cervical cancer prevalence, the Pap smear has drawbacks. False-positive and false-negative findings can happen, which can cause undue concern and follow-up treatments or delayed diagnoses. Some nations have included HPV testing in their screening programs to increase the precision of cervical carcinoma testing. High-risk HPV strains, which account for the primary cause of cervix cancer, can be detected by HPV testing for women between the ages of 30 and 65. The United States Preventive Services Task Force (USPSTF) advises co-testing with Pap smear and HPV screening every five years [10]. Comparing a Pap smear alone to co-testing was demonstrated to boost sensitivity in detecting cervical precancerous lesions [12].

Screening in Women With HIV

Cervical carcinoma is a significant concern for women suffering from the human immunodeficiency virus because of the synergistic consequences of HIV and high-risk human papillomavirus co-infection [18]. Due to HIV immunosuppression, there is an immense chance of developing cervical dysplasia, cancer, and a chronic HPV infection. In the next section, we examine several screening procedures for cervical cancer in women with HIV in more depth and speculate on potential screening and prevention issues [19].

HPV Immunization in Women With HIV

 In immunocompetent women, the human papillomavirus vaccine is beneficial in lowering the incidence of HPV infection and the ensuing cervical cancer abnormalities [20]. Studies have revealed that HIV-positive people, particularly those with advanced illnesses or low CD4+ T-cell counts, may have a reduced immunological response to vaccination [21]. Despite this, other studies have proven that HPV immunization remains helpful in this group, resulting in a decline in HPV-related lesions and enhancements to general health outcomes [22,23]. The significance of HPV vaccination before or early in the course of HIV infection, while the immune function is generally intact, must be emphasized. Including the HPV vaccine in routine HIV treatment, especially for young girls and women, can protect this susceptible population from cervical cancer [24].

HIV Screening Options

There is continuing research and debate over the best cervical cancer testing option for women infected with HIV. Especially among women with well-controlled HIV and increased CD4+ T-cell count, the standard method of yearly Pap screenings has come under fire for possibly overdiagnosing and overtreatment of transitory HPV infections [25]. Unfavorable outcomes of very aggressive therapy include cervical stenosis and pregnancy problems. By using high-risk HPV DNA screening as the primary testing procedure, it has demonstrated the likelihood of improving the sensitivity of cervical carcinoma detection in HIV-positive women [26]. This approach can identify women more likely to develop cervical dysplasia and direct appropriate follow-up and care by looking for high-risk HPV strains [13]. Cervical cytology and HPV DNA testing may improve screening precision and minimize pointless treatments.

Cervical Precancerous Lesions Management

HIV-positive women must carefully treat cervical intraepithelial neoplasia (CIN). HIV-related immune suppression can cause CIN lesions to become more aggressive, increasing the risk of progressing to invasive cervical cancer [27]. Therefore, careful observation and prompt treatment of precancerous lesions are essential in this group. Despite conventional treatment options, cold knife conization (CKC) and the loop electrosurgical excision procedure (LEEP) in HIV-positive women require more investigation. To provide evidence-based guidelines for treating CIN in women living with HIV, research on the long-term effects of these procedures in this particular group is crucial.

Screening Integration and Accessibility

By integrating cervical cancer screening services into HIV care settings, screening rates may increase, and fewer chances for prevention and treatment may be lost. Healthcare professionals can improve the accessibility of cervix cancer testing for women with HIV by offering screening during regular HIV clinic visits [28]. This integrated strategy can make prompt care coordination and follow-up for aberrant screening findings easier. Additionally, overcoming geographic and financial screening barriers is critical, particularly in areas with few resources where women are significantly affected by HIV and cervical cancer. Public health awareness programs and easily accessible, reasonably priced screening services can considerably help with early identification and better outcomes for women with HIV.

Prevention

Strategic Activities to Reach the 2030 Objectives (WHO)

The World Health Organization (WHO) has initiated a national program to eliminate cervical carcinoma, including strategic initiatives to meet the 90-70-90 objectives [12]. The extension should be part of national health strategies for societies to achieve universal healthcare. Implementation should be driven and steered by high-level political dedication and governance, assisted by cooperative alliances. Each evidence-based cervical cancer intervention has specific performance needs and presents particular difficulties. There will need to be more than biomedical and clinical treatments to meet the objectives. Many implementation difficulties are due to flaws in the healthcare system that frequently exist across countries with low or middle incomes, wherever the disease load is the highest [3]. Each individual's strategic activities must be tailored. The prevalence, mortality, and morbidity of cervical cancer may be influenced by a country's particular systemic flaws, level of preparedness, and other factors (such as sociocultural attitudes towards sexual orientation and preconceived notions about the illness and its prevention and intervention). Urban settings may require different methods for scaling up initiatives than remote and rural ones. Disparities in medical conditions require a concentrated approach among vulnerable or disadvantaged populations, including women with HIV. The worldwide eradication agenda demands that authorities collaborate with significant partners, including entrepreneurs and NGOs, and that impacted communities be meaningfully engaged and given authority. It can boost public sector productivity by leveraging management efficiencies seen in the commercial sector. Furthermore, social organizations may advocate the need for conveniently available, competitively priced, and widely accepted health goods and services to increase community awareness of cervical cancer prevention and management, especially for individuals at higher risk for the disease [12]. Survivors of cervical cancer should be empowered to act as champions for reducing stigma and informing women and girls of the advantages of immunization, testing, and therapy. The World Health Organization recommends an ongoing approach to an extensive strategy to eradicate cervical cancer to ensure long-lasting rewards.

Primary Cervical Cancer Prevention (HPV Immunization)

Teenage female immunization is the most efficient and long-lasting approach for reducing the incidence of cervix cancer. Launching and maintaining this strategy in all nations is crucial due to the significant long-lasting benefits of HPV immunization. Additionally, there is convincing and substantial proof that high HPV immunization rates result in widespread immunity, strengthening the population's protective impact [29]. According to current WHO recommendations, young teenage females and males between 9 and 15 years of age should take two vaccine doses to receive complete protection, and there should be at least six months between the first and second dosages [12]. Data showing safety after a single dose encouraged research, which will offer data for further adjustments to the schedule [30,31]. Geographical locations and income levels affect HPV vaccine coverage unevenly, with higher-income nations having better vaccination rates. The introduction of the HPV vaccine into national vaccination programs and the viability of present programs have both been severely hampered by high vaccine pricing and recent supply issues [12]. The launch of HPV vaccination programs must be followed by effective communication methods for advocacy and social mobilization to reinforce the vaccine's efficacy, safety, and advantages. It will help achieve substantial acceptability and continuous coverage. Customized approaches are necessary to combat the burgeoning anti-vaccine movement. HPV immunizations are accessible, adequate, and inexpensive; therefore, it is necessary to boost the effectiveness and reach of vaccination. Additionally, it is important to improve social mobilization and communication as well as improve vaccine delivery effectiveness through innovation. In addition to HPV vaccination, a comprehensive prevention strategy is shown below in Table 1.

**Table 1:** Strategic measures to get 90% HPV eradication (WHO) Human papillomavirus elimination by the World Health Organization [12].

Preventive Strategies		Actions for Implementations
1.	Obtain enough inexpensive HPV vaccines	To get over the shortage of vaccines, partners and the private sector will need to work together. More reasonable costs can be reached while preserving a viable HPV vaccination market through proper market-shaping initiatives.
2.	Improve vaccination quality and coverage	Increasing HPV vaccine coverage would need robust and long-term multi-sector delivery methods (such as school immunization programs) and innovative community-based tactics to target at-risk populations (such as adolescent females who are not enrolled in school). Monitoring systems or registers should track and improve availability and quality.
3.	Communication and community outreach	Social mobilization strategies will be required as HPV vaccination programs are initiated and escalated nationwide; it will be crucial to comprehend the social, cultural, societal, and other hurdles influencing how well the vaccination is received and used. Certain groups will require further participation to combat disinformation and overcome vaccination reluctance.
4.	Innovate to improve efficiency of vaccine delivery	Create new vaccine administration techniques that are more effective when new research and innovations on more effective and efficient methods of HPV vaccination become available.

Secondary Prevention of Cervical Lesions (Detecting and Treating Cervix Cancer)

By diagnosing and treating women with precancerous lesions, secondary prevention aims to decrease the incidence of and deaths caused by cervical carcinoma [32]. Further investigations (colposcopy and pathology) have been effectively able to meet cytology-based screening and disease treatment objectives when included in national programs with high coverage and adequate funding for monitoring patients [33]. Cytology-based programs have proven challenging to implement in underprivileged and middle-income countries, and when they have been, screening coverage is poor. In situations with limited resources, a secondary preventative strategy is to check the cervix with acetic acid visually and then treat it (examination and treatment). Even though it is relatively easy to set up, the provider significantly influences the quality of this type of visual examination, and its sensitivity varies. Because of the enhanced specificity, women who screened harmful for HPV must undergo a retest after an ideal interval of five years due to its substantial negative predictive value. Women should be able to self-sample to promote acceptability and access to services. HPV testing can be rapidly scaled up because of the already-in-use technical platforms now utilized in many countries to test for HIV, TB, and other illnesses. In an ideal world, HPV testing would replace other cervical cancer screening methods in all nations because of its high performance. Some techniques are supported by evidence for managing and evaluating women who test positive for HPV. Since treating women without access to screening is unethical, greater capacity for treating lesions found during cervix cancer testing is necessary. For women with precancerous lesions that are suitable for ablation, thermal ablation has just been included in the WHO treatment recommendations [34]. Market-shaping attempts to get reasonably priced, top-notch diagnostics and associated supplies will be given priority. The development of simple portable ablative treatment devices and artificial intelligence-based diagnostic tools presents tremendous prospects and brings the world closer to eliminating cervical cancer [35]. Table 2 below shows the strategy for 70% screening and 90% treatment for precancerous lesions.

**Table 2:** Strategic steps to attain 90% treatment of precancerous lesions and 70% coverage for screening (WHO guidelines) This table was taken from WHO guidelines on screening and treatment [12].

Screening and Treatment Strategies 70% & 90%		Steps to Achieving Screening and Treatment by 70% & 90%
1.	Recognize service access limitations and provide an enabling environment	It is paramount to have a thorough grasp of the structural, institutional, cultural and social barriers that prevent people from using services. Such information will guide the establishment of demand-generating tactics that are particular to the region and culturally suitable, acceptable, and accessible service delivery platforms. Women, in particular, need to be empowered and included in developing these vital programs to act as allies, combat stigma or misinformation, and assist individuals who require more care.
2.	Services for screening and therapy included in the primary healthcare package	Points of entry for women and girls to receive benefits include antenatal care, well-women clinics, HIV care and treatment clinics, and school-based health outreach programs. Patient inconvenience should be minimized, and opportunity costs should be minimized using people-centred referral procedures.
3.	Encourage the use of a screen-and-treat strategy	Nations will need to increase the locations where a single-visit screen-and-treat strategy might be to meet equality in health. Although single-visit screening and treatment strategies are only possible in some places, they should be used when necessary.
5.	Boost programs for quality assurance and laboratory capacity	Efficient, interconnected networks of laboratory services will make the most of scarce personnel and financial resources. Effective quality assurance programs are essential to guarantee that services adhere to the necessary standards. The delivery of services must include monitoring and training as crucial elements.

Advancement in cervical cancer treatment (tertiary prevention)

Immunotherapy

The body's immune system cannot inhibit the spread of cancer cells. Immune checkpoint inhibitors are newly developed drugs that "reset" the immune system. Immune checkpoint inhibitors have efficiently treated various cancers [36]. In recent years, clinical trials for these immune checkpoint inhibitors have been held because their value in treating cervical cancer has recently become known. There is presently only one immunotherapy drug approved to treat advanced cervical cancer. Numerous molecular characteristics including high malignant mutational burden (MMB), instability of microsatellite (IMS), increased expression of programmed death receptor 1 (PD-R1), and a high neoplasmic inflammatory state substantially support the justification for using immunotherapy in cervical cancer. Tumours have been recognized as a high malignant mutational burden (MMB) of cervical cancer [37]. The amount of somatic alterations per DNA mega-base has replaced neoantigen load as an independent predictor of immune checkpoint inhibitor therapy success, as recently discovered [38]. Still, clinical trials are ongoing to determine whether this or other immunotherapy medications would function better when combined with chemotherapy or chemoradiation [39].

Minimally Invasive Surgery (MIS): A Safe Surgery

There is controversy over the advantages of minimally invasive procedures over open surgery for surgically treating the initial stages of cervical cancer. MIS comprises robotic surgery and laparoscopic surgery. The laparoscopic procedure to cervical cancer (LACC) trial, the first randomized control trial, revealed unexpectedly troubling oncological results, displaying lesser disease-free and overall survival rates for women who underwent MIS than those who underwent open procedures [40]. From that point on, researchers conducted studies to refute these conclusions and reinstate the function of MIS in this context. Multiple retrospective investigations have shown that MIS has high survival rates comparable to open surgery. Minimally invasive procedures are less painful, require a shorter stay in the hospital, and have fewer complications. An up-to-date systematic analysis and meta-analysis comprising 3196 patients established the effectiveness of laparoscopic-assisted vaginal hysterectomy (LAVRH) in cases of the initial stages of cervical carcinoma. LARVH has no effect on disease-free survival (DFS) or overall survival (OS) in early-stage cervical carcinoma patients, and the outcomes of the open surgery group overlapped [41].

Treatment Combination for Cervical Cancer

The care of this type of tumour has been investigated using an approach combining chemotherapy with radiation, immunotherapy, and specific therapy because of the complications of cervical cancer. Integrating chemotherapy and immunotherapy can be a step forward in treating cervical carcinoma. Chemotherapy, combined with radiation, is highly known for inhibiting micro-metastasis and reducing cancer cell growth. Disease-free and overall survival are increased when vascular endothelial growth factor antibodies are added to conventional chemotherapy [42]. Compared to chemotherapy alone, the overall survival was improved when the monoclonal antibody (bevacizumab) medication was used with cisplatin and either paclitaxel or topotecan. Combining treatment approaches also lessens the toxicity and side effects of high dosages of immunotherapy. Nevertheless, despite these encouraging findings, more research remains necessary [43-45].

## Conclusions

In conclusion, a multifaceted strategy that includes immunization, screening, treatment, and palliative care constitutes a component of the public health strategy for eradicating and managing cervical cancer. This thorough analysis shows the successes, current initiatives, and potential directions in the global fight against cervical cancer, highlighting the need for a concerted effort to accomplish the long-term objective of eradicating this deadly but avoidable condition. Although there has been significant progress in preventing and controlling cervical cancer, issues remain to be resolved, including ensuring that all people have access to therapies as well as improving screening and treatment methods, and fortifying healthcare systems. In order to overcome these obstacles and progress toward eliminating cervical cancer, this analysis highlights the need for ongoing research, persistent political commitment, and cooperative efforts between governments, non-governmental organizations, and the corporate sector.
